# Dissociating Distractor-Filtering at Encoding and During Maintenance

**DOI:** 10.1037/a0036013

**Published:** 2014-02-10

**Authors:** Fiona McNab, Raymond J. Dolan

**Affiliations:** 1 School of Psychology, University of Birmingham, Birmingham, United Kingdom and Wellcome Trust Centre for Neuroimaging, University College London, London, United Kingdom; 2 Wellcome Trust Centre for Neuroimaging, University College London, London, United Kingdom

**Keywords:** distraction, distractor filtering, working memory, working memory capacity

## Abstract

The effectiveness of distractor-filtering is a potentially important determinant of working memory capacity (WMC). However, a distinction between the contributions of distractor-filtering at WM encoding as opposed to filtering during maintenance has not been made and the assumption is that these rely on the same mechanism. Within 2 experiments, 1 conducted in the laboratory with 21 participants, and the other played as a game on smartphones (*n* = 3,247) we measure WMC without distractors, and present distractors during encoding or during the delay period of a WM task to determine performance associated with distraction at encoding and during maintenance. Despite differences in experimental setting and paradigm design between the 2 studies, we show a *unique* contribution to WMC from both encoding and delay distractor performance in both experiments, while controlling for performance in the absence of distraction. Thus, within 2 separate experiments, 1 involving an extremely large cohort of 3,247 participants, we show a dissociation between encoding and delay distractor-filtering, indicating that separate mechanisms may contribute to WMC.

Working memory (WM) is important for a wide range of cognitive functions such as reasoning and language (Süβ, Oberauer, Wittmann, Wilhelm & Schulze, 2002; Baddeley, 2003). WM capacity (WMC) is reduced in many psychiatric and neurological disorders (Goldman-Rakic, 1994; Castellanos & Tannock, 2002) as well as with normal aging (Bopp & Verhaeghen, 2005). A possible functional architecture that determines the efficiency of WMC relates to filtering, which enables the discarding of irrelevant and storage of relevant information. It has been reported that storage-related parietal activity for distractors negatively correlates with WMC, consistent with the idea that participants with low WMC unnecessarily store distractors, whereas participants with high WMC filter them effectively and remember relevant information alone (Vogel, McCollough & Machizawa, 2005; McNab & Klingberg, 2008).

In studies of WM, distractors are sometimes presented during WM encoding (simultaneous with presentation of to be remembered stimuli), whereas in others distractors are shown during the delay period (when the WM stimuli are held in mind but no longer displayed). Previous work has shown that WM performance improves with sequential compared with simultaneous stimulus presentation, with the latter predicting improvement (Ihssen, Linden & Shapiro, 2010). Here we ask whether the efficiency of distractor filtering during the simultaneous presentation of targets and distractors differs from the efficiency of distractor filtering during the sequential presentation of targets and distractors, and whether they differ in terms of their contribution to WMC.

Distractor filtering processes at encoding and during delay have sometimes been described differently, for example the former seen as requiring a “selective gating mechanism” (Frank et al., 2001) whereas maintaining information in WM in the face of delay distraction is described as a process in which “the doors of perception close” (Bonnefond & Jensen, 2012). However, to the best of our knowledge a distinction between the two has not been experimentally probed and in some cases it has been assumed that encoding and delay distractor filtering rely on the same neural mechanism. For example, it has been argued that enhanced resistance to distraction seen in patients deficient in striatal dopamine (with distractors presented during the delay period) is not consistent with reports of striatal dopamine playing an important role in distractor filtering (referring to research in which distractors are presented during encoding, e.g., McNab & Klingberg, 2008; Cools et al., 2010). An involvement of striatal dopamine in encoding but not delay distractor filtering could account for such apparent inconsistency. Within this study we directly compare encoding and delay distractor filtering to challenge an assumption that the two processes are indistinct, and highlight a need to draw a distinction between encoding and delay distractor filtering in future research.

With two separate experiments we directly compare the contribution of encoding and delay distractor filtering to WMC to determine whether they each make a unique contribution to WMC. Such a finding would not only distinguish the two processes, and suggest that separate neural mechanisms may be involved, but also indicate that they represent two potential bases for WMC. Although their contribution to WMC has not been directly compared, research suggests that both encoding (e.g., Vogel et al., 2005; McNab & Klingberg, 2008) and delay distractor filtering predict WM performance. For example, participants with high WMC are reported to show greater accuracy for trials that include delay distractors (pictures of faces) compared with those with low WMC (Minamoto, Osaka & Osaka, 2010). Furthermore, greater activity seen in the fusiform gyrus in response to face distractors in participants with low compared with high WMC suggests distractors were not filtered as effectively.

Our two experiments differed in a number of substantive ways. For the laboratory experiment measures of WMC and distractor cost were obtained by presenting stimuli of a fixed set-size and estimating the number of items successfully remembered using the hit rate and false alarm rate (the K value; Vogel et al., 2005; Cowan, 2001). In the smartphone game the number of items to remember (WM load) in each condition increased in line with performance until the participant failed two successive trials. Performance was determined by the maximum load the participant successfully completed. The smartphone experiment also involved a much larger sample than the laboratory study, but time spent on the task had to be kept to a minimum and the environment in which the game was played could not be controlled. We hypothesized that, despite these differences, in both experiments distractor filtering at encoding and at delay would *uniquely* predict WMC.

## Laboratory Study

### Method

#### Participants

Twenty-three healthy participants gave informed consent to participate in the laboratory study, which was approved by the University College London Ethics Committee. Two participants failed to complete the study, leaving 21 (8 females, ages 20–29, right-handed).

#### Experimental design and task

Data were collected during the placebo condition of a drug study, the results of which are not reported here. Participants performed the experiment twice, 7 days apart. On one day they received levodopa (150 mg + 37.5 mg benserazide), and on the other day they received placebo (500 mg ascorbic acid). The order was counterbalanced such that 11 received levodopa on their first visit and 10 received levodopa on their second visit. The study was double-blind. Participants began the WM tasks approximately 2 hours and 15 minutes after receiving either levodopa or placebo, and after completing a MEG study of decision-making, the results of which are not reported here. Only data from the placebo condition are considered here, and as we used a repeated measures design we consider that differences in previous exposure to the task could not have affected the results.

The experiment consisted of the following computer-based WM tasks. The experiment was divided into three sections, with the order of the three sections counterbalanced across participants. The three sections are described below.

#### Working memory capacity (WMC)

**Figure 1 fig1:**
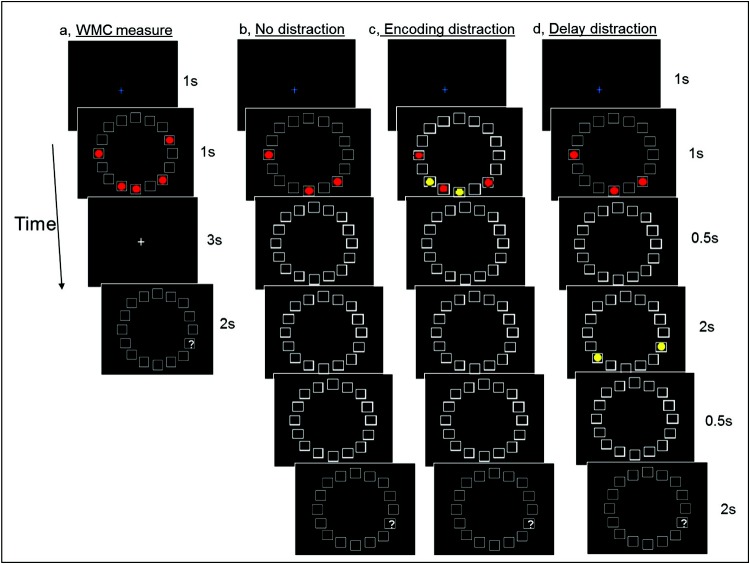
a) The figure illustrates the task used to obtain an estimate of WMC in the absence of distraction. Participants were asked to remember the positions of five red target circles displayed for 1 s followed by a delay period of 3 s (during which a white fixation cross was shown) and respond to a probe stimulus (a question mark presented for 2 s) which asked participants to indicate whether a red target circle had been shown in the position indicated (a yes/no response). Targets were positioned such that no more than two targets were in adjacent positions. The probe was shown either in or adjacent to one of the target positions. Forty trials were given, with half of the probes presented in a target position and half presented in a position adjacent to a target position. b through d) The three conditions used to obtain estimates of distractor filtering ability at encoding and delay. Target circles were displayed for 1 s, with no more than two of the targets in adjacent positions. The circular grid remained on the screen throughout each trial. A probe (a white question mark) was shown either in or adjacent to one of the target positions for 2 s, 3 s after the target stimuli had disappeared. b) In the “no distraction” condition participants were required to remember the locations of three red target circles, and no distractors appeared. c) In the “encoding distraction” condition two yellow distractor circles were shown together with the three red target circles. One of the yellow distractor circles was always in a position adjacent to a target position. d) In the “delay distraction” condition two yellow distractor circles were shown during the delay period, 0.5 s after the red target circles had disappeared. One of the yellow distractor circles was always in a position adjacent to a target position. Participants were asked to remember the positions of only the red target circles, and give a yes/no response to indicate whether the probe was in a position that had been occupied by a red target circle.

To obtain a measure of WMC participants were asked to remember the positions of five red circles (targets) presented simultaneously on a circular grid (Figure 1a).[Fig-anchor fig1]

#### Distractor-filtering at encoding and during the delay

Measures of accuracy with and without distraction were obtained from a task in which 3 red circles (target stimuli) were presented simultaneously. In the “no distraction” condition (Figure 1b) no yellow distractor circles were shown. In the “encoding distraction” condition (Figure 1c) two yellow distractor circles were presented together with the three target circles. In the “delay distraction” condition (Figure 1d) two yellow distractor circles were presented after the three target circles had disappeared. Participants were asked to remember the positions of the red target circles, ignore the yellow distractor circles, and indicate with a button press whether the probe was in one of the target positions. Trials were presented in two blocks, with 60 trials in each. The trials in each block were divided equally between the three conditions, and presented in a pseudorandom order. Half of the trials in each condition required a “yes” response, and in both the encoding and delay distraction conditions, half of the trials that required a “no” response had the probe presented in the position of a distractor.

#### Forward and backward span tasks

Participants performed one backward span task and two forward span tasks, as described previously (McNab et al., 2009), the results of which are not reported here.

#### Data analysis

WMC was estimated with the *K* value, estimating how much information can be stored in working memory, using a standard formula (Vogel et al., 2005; Cowan, 2001); *K* = *S* (*H* − *F*), where *K* is the WMC, *S* is the array size, *H* is the observed hit rate, and *F* is the false alarm rate. This uses the false alarm rate to correct for guessing and assumes that if *K* items can be held in WM, from an array of *S* items, the probed item would have been one of those held in memory on *K*/*S* of trials, so that performance will be correct on *K*/*S* of the trials.

For each participant we calculated four *K* values; one for the WMC task, one for the no distraction condition, one for the encoding distraction condition, and another for the delay distraction condition.

To determine whether the effects of encoding and delay distraction on performance uniquely predict WMC, we performed a hierarchical regression analysis. In this hierarchical regression, performance in the no distractor condition was used to predict WMC, and then performance in both the encoding and delay distractor conditions was added to the model. *R*^2^ change between the two models was used to assess the variability in WMC that could be explained by distractor filtering in general. Once all predictors were in the model we examined the unique contributions of encoding and delay distraction while controlling for WM performance in the other conditions.

All statistical analyses were performed using IBM SPSS Statistics 20. For all correlations, *p* values were determined with two-tailed analyses.

### Results

Values for WMC for each of the conditions in the two studies are shown in Table 1, and zero-order correlations are shown in Table 2. As shown in Table 1, although *K* values for the measure of WMC (load 5, no distractors) were lower than for each of the load 3 conditions, when comparing performance across the load 3 conditions, performance declined with the inclusion of distractors.[Table-anchor tbl1][Table-anchor tbl2]

**Figure 2 fig2:**
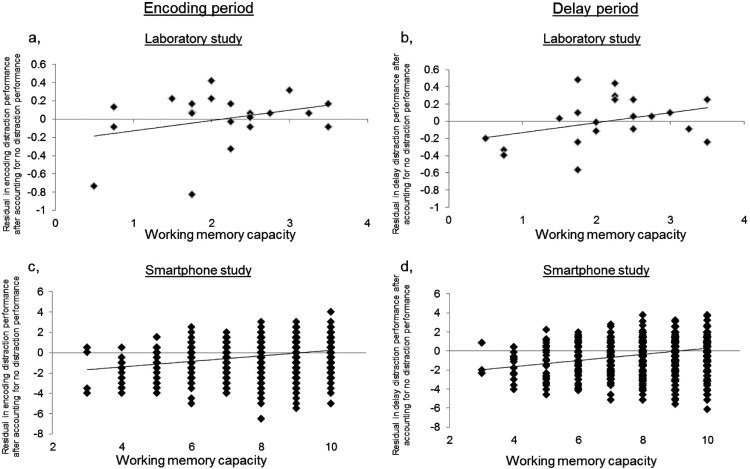
Positive associations between WMC and the residual after accounting for no distraction performance in a) the encoding distraction condition and b) the delay distraction condition for the laboratory study, and between the short delay condition and the residual after accounting for no distraction performance in c) the encoding distraction condition and d) the delay distraction condition for the smartphone study. The positive associations indicate that greater distractor filtering performance is associated with greater WMC.

As shown by Table 3, all predictors together significantly predicted WMC (adjusted *R*^2^ = 0.39, *F*_3,17_ = 5.29, *p* = .009). Over and above the no-distractor condition, an additional 28% of the variability (*p* = .025) in WMC was explained by adding both encoding and delay distractor performance to the model in which no distraction performance was used to predict WMC. Both K values at encoding and delay distraction conditions significantly and uniquely predicted WMC while controlling for performance in the other two conditions (encoding distraction: standardized β = 0.616, *p* = .038; delay distraction: standardized β = 0.615, *p* = .025, Figure 2a and 2b). The positive associations indicate that greater distractor filtering performance is associated with greater WMC. Furthermore, no significant correlation was seen between encoding distraction and delay distraction performance (partial correlation controlling for WM no distraction, *r* = −0.211, *p* = .372), in keeping with separate mechanisms being involved with distractor-filtering at encoding and delay.[Table-anchor tbl3][Fig-anchor fig2]

## Smartphone Study

### Method

#### Participants

Data from participants aged 18–29 years were considered. Data were excluded from participants who failed at the easiest level of any of the conditions (i.e., failed two consecutive trials of WM load 2). Following these exclusions, data from 3,247 participants remained for analysis.

#### Experimental design and task

This smartphone game formed part of the “The Great Brain Experiment,” which is funded by the Wellcome Trust (*http://thegreatbrainexperiment.com*). The working memory game involved six conditions, four of which are described here. Participants were asked to remember the positions of red circles that appeared on a 4 × 4 grid for 1 s. At the end of each trial they were presented with an empty grid and asked to press on the grid positions in which red circles had appeared. In three of the conditions there was a delay period of 1 s during which time an empty grid was shown, after the red circles had disappeared and before participants could make their response. In one of these conditions (“no distraction”) only red circles were displayed. In another (“encoding distraction), two yellow distractor circles were shown together with the red circles. In the third of these conditions (“delay distraction”), two yellow distractor circles were displayed during the delay period. In the “short delay” condition, only red circles were shown and participants could begin making their response immediately after the circles had disappeared. For each condition the number of red circles (WM load) increased with performance (one red circle added each time two successive trials were answered correctly) until the participant failed two successive trials of a condition (from which point the game continued without that condition) or the game timed-out.

#### Data analysis

Although participants were able to play the game multiple times, we only considered data from their first play, to ensure that practice effects could not influence the results. Performance in each condition was measured as the last WM load at which two successive trials were answered correctly. To determine whether encoding and delay distraction performance uniquely predicted WMC, when controlling for performance in the absence of distractors, we again performed a regression analysis. We used the score from the “short delay” condition as our measure of WMC (the dependent variable) because it did not involve any distractors. With hierarchical regression, performance in the no distractor condition was used to predict WMC, and then performance in both the encoding and delay distractor conditions was added to the model. Again, *R*^2^ change between the two models was used to assess the variability in WMC that could be explained by distractor filtering in general. Once all predictors were in the model we examined the unique contributions of encoding and delay distraction while controlling for WM performance in the other conditions.

### Results

Values for WMC for each of the conditions in the two studies are shown in Table 1, and zero-order correlations are shown in Table 2. Performance was lower for both the encoding and delay distraction conditions compared with both our measure of WMC and the no distraction condition.

As anticipated, the estimates of WMC are very different for the two studies, most likely because of the very different ways in which the measures were obtained (*K* values for the laboratory study, maximum level reached for the smartphone study).

As shown by Table 3, all predictors together significantly predicted performance during the short delay condition (adjusted *R*^2^ = 0.290, *F*_3,3243_ = 442.079, *p* < .001). Over and above the no-distractor condition, an additional 13% of the variability (*p* < .001) in WMC was explained by adding both encoding and delay distractor performance to the model in which no distraction performance was used to predict WM performance in the short delay condition. Both performance in encoding and delay distraction conditions significantly and uniquely predicted WM performance in the short delay condition, while controlling for performance in the other two conditions (encoding distraction: standardized β = 0.263, *p* < .001; delay distraction: standardized β = 0.230, *p* < .001), in keeping with separate mechanisms for distractor-filtering at encoding and delay making a unique contribution to WMC. The positive associations indicate that greater distractor filtering performance is associated with greater WMC. This time, a significant correlation was seen between performance on encoding and delay distraction conditions (partial correlation controlling for performance on the no distraction condition, *r* = .315, *p* < .001), indicating that although they make a unique contribution to WMC, their performance was not completely unrelated in the context of the smartphone study. Possible explanations for this are considered in the discussion.

Similarly, when we used performance on the short delay condition as the baseline condition (as a predictor variable, and performance on the WM no distraction condition as our measure of WMC (the dependent variable), both encoding and delay distraction performance significantly and uniquely predicted WMC (adjusted *R*^2^ = 0.294, *F*_3,3243_ = 451.38, *p* < .001, for encoding distraction performance standardized β = 0.285, *p* < .001; for delay distraction performance standardized β = 0.214, *p* < .001). Again there was a significant correlation between performance on the encoding and delay distraction conditions (partial correlation controlling for performance in the short delay condition, *r* = .316, *p* < .001).

Because the maximum possible score for each condition was 10 (as a result of the game timing-out), to ensure that our results were not a consequence of ceiling effects we repeated the analysis only considering data from participants who scored less than 10 in the no distraction and the short delay conditions (*n* = 986). Regression analysis again showed a unique contribution from both encoding and delay distraction to performance in the short delay condition (adjusted *R*^2^ = 0.234, *F*_3,982_ = 101.26, *p* < .001, encoding distraction: standardized β = 0.242, *p* < .000; delay distraction: standardized β = 0.233, *p* < .000). Using a partial correlation (controlling for performance on the no distraction condition) we again found a significant correlation between performance on encoding and delay distraction conditions (*r* = .324, *p* < .001).

## Discussion

In line with previous work in which encoding distractor-filtering has been identified as a potential basis for WMC (Vogel et al., 2005), in two studies we found that participants with greater WMC were less affected by distraction presented during encoding, indicating more effective distractor-filtering. We extend this work by comparing performance associated with encoding and delay distraction, while controlling for performance in the absence of distraction, in the same individuals. We used one standard laboratory experiment with data from 21 participants, and a large-scale study with data collected from 3,247 participants using smartphones. For both studies multiple regression analysis revealed that both encoding and delay distractor-filtering significantly predicted WMC, each accounting for *unique* variance in WMC, highlighting a distinction between encoding and delay distractor filtering.

The unique contributions we observe from encoding/delay distractor filtering in predicting WMC provide tentative evidence that encoding and delay distractor-filtering make separate contributions to WMC, and that separate mechanisms for encoding and delay distractor-filtering might contribute to WMC. The fact that we see such associations when controlling for WM performance in the absence of distraction argues against the alternative account in which those with high WMC have extra WM resources available to successfully encode the distractors. Such extra capacity would only be available if baseline performance was at ceiling. Because no participant reached the maximum of *K* = 5 in the WMC condition of the laboratory study, and we have replicated the results of the smartphone study while excluding participants who reached the maximum level (level 10) in the no distraction or short delay conditions, our results are not explicable by mere ceiling effects. Furthermore, in the case of encoding distractor filtering, preparatory brain activity associated with distractor filtering, before the onset of target stimuli has been shown to predict WMC and the unnecessary storage of distractors (McNab & Klingberg, 2008) indicating that, at least for encoding distraction, preparation to filter distractors makes a contribution to subsequent WM performance.

The laboratory experiment and the smartphone study differed in a number of ways besides the size of the sample. For example, in the laboratory experiment we used the *K* value as a measure of WM performance, whereas for the smartphone study, estimates of WM performance were obtained by increasing WM load and identifying the load at which the participant failed. The *K* value can be obtained by averaging over many trials and takes into account guessing, so may be a more precise measure, whereas increasing load to find the participant’s limit is more suited to a game format, and may therefore be more motivating. As anticipated, the different measures of WMC yielded very different values of WMC in the two studies (see Table 1). Many other differences between the two studies may have contributed to this difference. For example, stimuli were presented on smaller screens in the smartphone study, in a rectangular grid (perhaps enabling easier labeling of different grid positions, and easier chunking compared to the circular grid used in the laboratory study) and participants were encouraged to play competitively. Furthermore, we were unable to control the environment in which the smartphone game was played. Regardless of these differences, we find a unique contribution from both encoding and delay distraction cost to WMC with both approaches. The different encoding and delay distractor durations used in the laboratory study (1 s and 2 s, respectively) and the smartphone study (1 s and 1 s, respectively) indicate that differences in distractor duration do not affect the separate contribution of encoding and delay distractor filtering to WMC that we observe in both studies.

The positive correlation between encoding and delay distraction observed in the smartphone study suggests that although we find evidence for unique contributions from encoding and delay distraction, shared variability may also exist. A common mechanism may also contribute differently to conditions of encoding and delay distraction, for example being sustained for one but not the other. Another difference between the laboratory and the smartphone study was a significant positive correlation between encoding and delay distraction cost in the latter but not the former, when controlling for performance in the absence of overt distractors. Although this does not affect our main result (the significant and unique contribution made by encoding and delay distraction to WMC in the two studies), the difference is intriguing and indicates that encoding and delay distraction performance is not entirely unrelated under certain conditions. One possibility is that a difference in WM load may have affected their association. In the laboratory study WM load was held constant at load 3, whereas WM load in the smartphone study increased in line with performance, so that participants were exposed to higher load trials. Another possibility is that in more distracting environments the ability to ignore encoding and delay distraction becomes more closely associated. Whether there is a closer association between encoding and delay distractor filtering at higher WM loads or in more distracting environments, and the mechanisms responsible for this, are questions for future research.

Separate mechanisms for distractor-filtering at encoding and during the delay could resolve an apparent inconsistency highlighted by Cools et al. (2010). They observed impaired backward span performance in patients with PD off medication, which is presumably a result of deficient striatal dopamine, yet enhanced distractor-filtering. Similarly, Mehta et al. (2004) observed a task set shifting impairment but an improvement in resistance to distraction after sulpiride, a D2 receptor antagonist that specifically affects the striatum (Mehta et al., 1999; Mehta et al., 2003). Cools et al. (2010) acknowledge that distractor filtering has also been linked to the functions of the basal ganglia (McNab & Klingberg, 2008; Gruber et al., 2006), revealing an apparent inconsistency. However, both Mehta et al. (2004) and Cools et al. (2010) presented distractors during the delay period, whereas the study which linked distractor-filtering to the striatum presented distractors only during encoding (McNab & Klingberg, 2008). If encoding, but not delay distractor filtering, involves the striatum this apparent inconsistency would be resolved.

A model for selection in WM has been proposed based on disinhibitory gating in the motor domain, putatively involving the basal ganglia selectively initiating the storage of new memories by “unlocking the gate” to memory maintenance in the frontal cortex (Frank et al., 2001). In this way frontal memory representations can be rapidly updated depending on task-relevance. One possible account for our findings is that a striatal gating mechanism is specific to encoding, but not delay, distractor-filtering. In the case of delay distractor filtering, the gate allowing access to WM has presumably already been “locked,” and an alternative mechanism is then required to preserve a frontal memory representation in the face of distraction. This may take the form of an increase in the power of alpha oscillations which serve to close “the doors of perception,” allowing effective delay distractor filtering (Bonnefond & Jensen, 2012). It also seems likely that delay distractor-filtering involves the frontal cortex but not the striatum as during delay distraction, Cools et al. (2007) observed that activity in lateral frontal cortex, but not the striatum, was potentiated by the dopamine agonist Bromocriptine. Single cell recordings have shown that some PFC neurons create spatially tuned delay activity as a result of delay distraction (Artchakov et al., 2009). Similarly, middle frontal cortex/dorsolateral prefrontal cortex (DLPFC) and inferior frontal gyrus (IFG) have been specifically implicated in delay distractor-filtering (Dolcos, Miller, Kragel, Jha & McCarthy, 2007; Sakai, Rowe & Passingham, 2002) with DLPFC linked to the enhancement of relevant information in the presence of distraction (Feredoes, Heinen, Weiskopf, Ruff & Driver, 2011) and IFG linked to the inhibition of interference (Dolcos et al., 2007; Aron, Robbins & Poldrack, 2004). Furthermore, Minamoto et al. (2010) observed that participants with high compared with low WMC showed greater activity in left middle frontal gyrus during delay distraction, greater accuracy on delay distraction trials, and less activity in sensory cortices (fusiform gyrus) during distractor presentation. We propose that the evidence suggests top-down modulation from the left middle frontal gyrus provides more effective delay distractor-filtering in participants with high WMC, and is a possible neural basis for the delay distraction-filtering mechanism we identify. A frontal delay distractor-filtering mechanism would also fit with the data of Cools et al. (2010) and Mehta et al. (2004) if compensatory upregulation of frontal dopamine underlies their findings of improved delay distractor-filtering in PD and with sulpiride, respectively.

As well as the basal ganglia, middle frontal cortex is implicated in encoding distraction filtering (McNab & Klingberg, 2008). Toepper et al. (2010) observed ventrolateral and dorsolateral PFC activity during a Corsi Block Tapping task, and greater DLPFC activity in a version where distractors were added “during encoding.” Based upon this finding it was suggested that DLPFC is involved with higher-level executive processes including both inhibition of spatial distraction (Toepper et al., 2010). However, in this study spatial locations to be remembered were presented sequentially, so that a distractor presented during the encoding of the second stimulus in the sequence, for example, would have also acted as a delay distractor for the first stimulus in the sequence. Therefore, it is difficult to attribute enhanced DLPFC activity they observe specifically to the effects of encoding distraction. Further work is needed to establish whether encoding and delay distractor-filtering involve different frontal mechanisms, and to understand the basis for the dissociation we observe between encoding and delay distractor-filtering.

It is clear that there are a number of differences between encoding and delay distractor conditions. For example, encoding distractors may group with the targets given their common onsets, whereas the delay distractors have a sudden onset after presentation of the targets, which may capture attention. There may also be differences in arousal during the different stages of the WM task. Further work is needed to manipulate and control such factors to decipher their relative contribution to WMC and the neural mechanisms responsible for overcoming their distracting effects.

The evidence we provide, suggesting separate mechanisms for encoding and delay distractor-filtering, and their separate contributions to WMC, could have implications for understanding the basis of WMC impairments, for example in schizophrenia and healthy aging. In these groups distractor-filtering has tended to be studied with distractors presented during a delay, or between the sequential presentation of the stimuli that are to be remembered, preventing a clear distinction between encoding and delay distractor-filtering (e.g., Anticevic, Repovs, Krystal & Barch, 2012; Cameron et al., 2002; Gazzaley, Cooney, Rissman & D’Esposito, 2005). Furthermore, cognitive training can lead to improvements in WMC (Klingberg, Forssberg & Westerberg, 2002; Dahlin et al., 2008) with good evidence to suggest that distractor-filtering can be trained (Melara, Rao & Tong, 2002). By identifying the extent to which encoding or delay distractor-filtering is impaired in a certain individual, it may be possible to tailor the cognitive training to the individual and increase improvements.

In conclusion, with two separate studies (one a standard laboratory experiment with data from 21 participants, and the other a large-scale study with data collected from 3,247 participants using smartphones), we provide evidence for a dissociation between encoding and delay distractor-filtering, with both separately contributing toward WMC. These therefore represent two potential bases for WMC.

## Figures and Tables

**Table 1 tbl1:** Mean Working Memory Capacity (K) for Each Condition for Both the Laboratory and Smartphone Studies

Study	WMC measure	No distraction	Encoding distraction	Delay distraction
Laboratory study	2.13 (± 0.84)	2.79 (± 0.21)	2.59 (± 0.47)	2.68 (± 0.38)
Smartphone study	9.15 (± 1.14)	9.26 (± 1.05)	9.12 (± 1.15)	8.74 (± 1.42)
*Note*. For the laboratory study, “WMC measure” refers to the task used to estimate WMC in the absence of distraction (load 5). For the smartphone study, “WMC measure” refers to the “short delay” condition.				

**Table 2 tbl2:** Results of Pearson Correlations (R) Between Each Variable in Each of the Two Studies

Study	WMC measure	No distraction	Encoding distraction
Laboratory study			
No distraction	0.40**	—	—
Encoding distraction	0.45**	0.46**	—
Delay distraction	0.43**	0.42**	0.45**
Smartphone study			
No distraction	0.45*	—	—
Encoding distraction	0.54*	0.76**	—
Delay distraction	0.57**	0.70**	0.43
* *p* < .05. ** *p* < .01.			

**Table 3 tbl3:** Results of the Hierarchical Regression in Which Model 1 Predicts WMC Using Performance From the No Distraction Condition, and Model 2 Predicts WMC Using Performance From No Distraction Condition Together With Both the Encoding Distraction and Delay Distraction Conditions

Study	Model	Predictor	*R*^2^ change	Standardized beta	*p* value	Partial correlation
Laboratory study	1	No distraction	0.20	0.45	0.04	
	2	No distraction	0.28	−0.45	0.21	−0.30
		Encoding distraction		0.62	0.03	0.48*
		Delay distraction		0.62	0.04	0.51*
Smartphone study	1	No distraction	0.16	0.40	0.00	
	2	No distraction	0.13	0.18	0.00	0.18***
		Encoding distraction		0.26	0.00	0.25***
		Delay distraction		0.23	0.00	0.23***
*Note*. The results of partial correlations are also shown for each of the predictors in Model 2, showing the extent to which they correlate with WMC, whilst controlling for the other two predictor variables.						
* *p* < .05. ** *p* < .01. *** *p* < .001.
